# Estimating the Economic Impact of Respiratory Syncytial Virus and Other Acute Respiratory Infections Among Infants Receiving Care at a Referral Hospital in Malawi

**DOI:** 10.1093/jpids/piaa157

**Published:** 2020-12-21

**Authors:** Ranju Baral, Ivan Mambule, Elisabeth Vodicka, Neil French, Dean Everett, Clint Pecenka, Naor Bar-Zeev

**Affiliations:** 1 Center for Vaccine Innovation and Access, PATH, Seattle, Washington, USA; 2 Clinical Research Programme, Malawi Liverpool Wellcome Trust Clinical Research Programme, Blantyre, Malawi; 3 Institute of Infection Veterinary and Ecological Sciences, University of Liverpool, Liverpool, UK; 4 Centre for Inflammation Research, University of Edinburgh, Edinburgh, UK; 5 International Vaccine Access Center, Johns Hopkins Bloomberg School of Public Health, Baltimore, Maryland, USA

**Keywords:** acute respiratory infections (ARIs), costs, cost per episode, household costs, respiratory syncytial virus (RSV)

## Abstract

**Background:**

Respiratory syncytial virus (RSV) is a leading cause of respiratory illness among infants globally, yet economic burden data are scant, especially in low-income countries.

**Methods:**

We collected data from 426 infants enrolled in the Queen Elizabeth Central Hospital respiratory disease surveillance platform to estimate the household and health system costs of managing RSV and other respiratory pathogens in Malawian infants. Total household cost per illness episode, including direct and indirect costs and lost income, was reported by parents/guardians at the initial visit and 6 weeks post discharge. The total cost to the health system was based on patient charts and hospital expenditures. All-cause acute respiratory infections (ARIs) and RSV costs for inpatient and outpatients are presented separately. All costs are in the 2018 US Dollar.

**Results:**

The mean costs per RSV episode were $62.26 (95% confidence interval [CI]: $50.87-$73.66) and $12.51 (95% CI: $8.24-$16.79) for inpatient and outpatient cases, respectively. The mean cost per episode for all-cause ARIs was slightly higher among inpatients at $69.93 (95% CI: $63.06-$76.81) but slightly lower for outpatients at $10.17 (95% CI: $8.78-$11.57). Household costs accounted for roughly 20% of the total cost per episode. For the lowest-income families, household cost per inpatient RSV episode was about 32% of total monthly household income.

**Conclusions:**

Among infants receiving care at a referral hospital in Malawi, the cost per episode in which RSV was detected is comparable to that of other episodes of respiratory illnesses where RSV was not detected. Estimates generated in this study can be used to evaluate the economic and financial impact of RSV and acute respiratory illness preventive interventions in Malawi.

## Teaser

Economic burden data for infant acute respiratory illness are scant in low-income countries and almost nonexistent for respiratory syncytial virus (RSV). We found the cost per RSV episode to be substantial, yet comparable to other respiratory illnesses in Malawi.

Acute respiratory infections (ARIs) are leading causes of childhood illness and death worldwide, accounting for roughly 15% of all deaths under the age of 5 globally [1]. Viruses constitute more than 60% of ARI etiology [2]. Respiratory syncytial virus (RSV) is a common cause of childhood ARIs, accounting for roughly 33 million cases and 118 000 deaths worldwide annually [3].

In Malawi, an estimated 11% of all childhood deaths are attributed to ARIs [4]. Further, surveillance data suggest that more than 54% of pediatric severe ARIs are due to viral respiratory infections, with RSV alone accounting for about 13% of hospitalized ARIs [5, 6]. RSV is particularly severe among infants, and those younger than 6 months of age account for a substantial portion of the disease burden [3].

While the overall African ARI epidemiology and disease burden data are sparse, Malawi has a well-established respiratory surveillance system and has identified notable disease burden. However, the economic impact of ARIs is not well documented, quantified, or understood—a major challenge for effective management of such illnesses. In Malawi, the direct medical expenses in public health facilities are provided free of cost. The health facilities are funded through the government district health offices, and people access all these services for free.

In this study, we evaluated the economic burden of infant RSV and other ARIs on Malawian families and on the healthcare system. We estimated the direct and indirect health care and household costs of managing RSV and other ARIs among both hospitalized (inpatient) and nonhospitalized (outpatient) infants. The cost estimates generated in this study can provide useful insights into the extent of economic losses due to RSV, inform cost-effectiveness analyses assessing the potential value of disease prevention strategies, and help global and country decision-makers formulate policy for the introduction and financing of preventive interventions when available.

## METHODS

### Study Area and Population

The costing study was nested within a large ARI surveillance program at the Queen Elizabeth Central Hospital (QECH) in Blantyre, Malawi. QECH is a large tertiary referral hospital serving predominantly urban and peri-urban areas of Blantyre District but with a catchment area that extends to all of southern Malawi.

Infants who presented with ARIs and were admitted to hospital and ambulatory wards were screened, received nasopharyngeal and oropharyngeal swabs and chest X-rays, and were recruited to participate in the surveillance program. Infants enrolled in the surveillance program and living in Blantyre were included in the costing study. Exclusion criteria included the following: a previous hospitalization for ARI treatment lasting more than 24 hours or within 14 days of presentation to QEHC; being admitted for more than 72 hours prior to screening; and moribundity or oncological or terminal illness.

### Data Collection

Cost data were collected between May 2015 and September 2016. A trained research assistant administered a standard survey questionnaire to the primary caregiver at the hospital. Questions included demographics, socioeconomic status, history of illness prior to presentation at the facility, and any prior costs incurred due to illness. Medical records for hospitalized patients were reviewed daily for detailed information on laboratory investigations, procedures performed, and drugs dispensed (including mode of administration, dose, route, amount, and duration of use). Outpatient data came from government-issued patient-held medical records. Study staff conducted follow-up home visits 6 weeks after the initial visit, to gather information about subsequent costs associated with the illness episode. Participants were also asked if they made additional facility visits for related symptoms after the initial visit, and if so, what the associated costs were. We did not gather follow-up posttreatment costs data from the deceased infant household.

#### Cost to the Health System

All costs incurred for patient care while patient is at the hospital are incorporated into hospital budget and are covered by the national government. Direct medical costs paid by the health system include drugs, laboratory tests and procedures, staff, and facilities (defined below). Unit costs associated with each were gathered from the QECH hospital using direct itemized costing of actual expenditures.

##### Drug Costs.

Unit cost of drugs was based on QECH’s acquisition costs per procurement records (see Supplementary Appendix Table 1). We assumed injectables were only used once, incurring the full cost of the product, regardless of wastage. We accounted for the full cost of drugs administered or dispensed, without considering adherence post dispensation.

##### Laboratory and Investigation Costs.

 To calculate the cost of laboratory tests and investigations, we multiplied the cost per unit test by the number of tests/investigations performed. Unit cost for the laboratory procedures/investigations set by the hospital (see Supplementary Appendix Table 2) was used to estimate the lab/procedure costs per patient. We excluded the cost of laboratory tests and investigations performed as part of clinical research, as these are likely not reflective of costs incurred during standard clinical care.

##### Staff Costs.

 Staff costs per bed day for inpatient care were based on the aggregated daily salary of all staff involved in the management and care of children with ARIs divided by the number of bed days in respective wards. Staff costs per bed day were then multiplied by the length of a patient’s hospital stay. Staff costs for outpatient care were based on the sum of daily salaries for all staff working in the ambulatory and emergency care department divided by the average number of outpatient visits per day.

##### Facility Costs.

Facility costs were calculated by dividing QECH’s annual facilities expenses by the total number of hospital bed days, then multiplying by the length of stay for inpatients. Facility expenses included administrative/operational expenses on items, such as procurement, stationery, uniforms, bedding and linens, kitchen, lighting, heating, telephone, water and sanitation, security, fuel, and vehicle maintenance. We assumed a half-day facility cost per outpatient case.

#### Cost to the Household

 Cost to the household includes both direct and indirect costs incurred during the episode of illness. Categories include prehospital visit costs, index visit costs, and posthospital visit costs.

##### Prehospital Visit Costs.

These include direct medical costs (such as consult, drugs, and laboratory tests) incurred by the household at other health facilities, prior to the index visit.

##### Index Visit Costs.

These include any direct medical costs not covered by the health system and direct nonmedical costs, such as transportation and subsistence for the patient, guardian, and visitors. Index visit costs also include indirect costs, such as a parent’s/guardian’s self-reported income lost while taking care of the patient.

##### Posthospital Visit Costs.

These include direct medical costs incurred by the household after hospital discharge. Posthospital visit costs were not collected for participants who died before the 6-week follow-up visit.

Cost data were collected in Malawi Kwacha (MWK) and inflation-adjusted to the 2018 MWK units using Malawi’s inflation rates [7]. Costs were then converted into the 2018 US dollar (USD) using the midyear official exchange rate (1 USD = 732.33 MWK) [8].

### Sample Size and Molecular Testing

A minimum sample size of 80 RSV-positive cases was required to provide a precision of ± 10% around the mean cost estimate for RSV episodes, at 0.5 coefficient of variation. Assuming RSV accounts for 20% of all ARI, we aimed to recruit 440 infants with ARI [9]. RSV etiology was confirmed through real-time multiplex polymerase chain reaction testing of nasopharyngeal swabs. We conducted RSV-specific and all-cause ARI analysis separately and by inpatient vs outpatient status.

### Ethics

Malawi National Health Science Research Committee (#1073) provided ethics approval for this study. All study subjects’ guardians provided written consent.

## RESULTS

### Participant Characteristics

**Figure 1.**
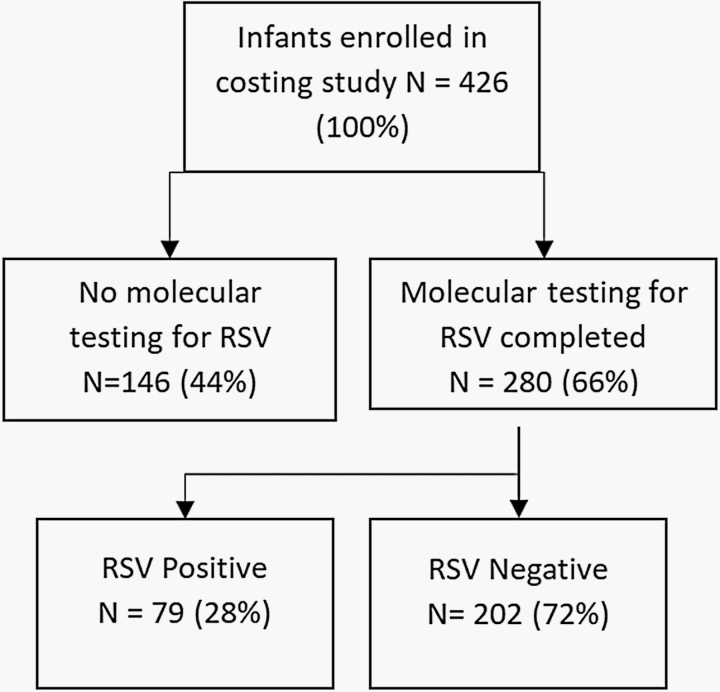
Patient flow diagram.

A total of 426 infants were included in this study (Figure 1) Fifteen (3.5%) of the included children were not alive at the 6-week follow-up home visit. The mean age of participants was 5.9 months at the time of enrollment (standard deviation [SD] 3.28), and a majority were male (63%). Inpatients constituted 75% of all cases, reflecting a higher proportion of the hospitalized cases due to their enrollment in the surveillance platform.

Due to funding constraints, only 280 (66%) swabs were tested to confirm RSV etiology. Among those tested, 78 (28%) were RSV positive. Of the 280 patients who underwent molecular testing to confirm RSV etiology, 265 (95%) patients (including all RSV confirmed positive cases) were also tested for detection of other non-RSV pathogens. Of the 78 RSV confirmed patients, 67 (86%) were detected with at least 1 non-RSV pathogen. No other non-RSV pathogen was detected among 11 (14%) of RSV-positive cases. Distribution of other non-RSV pathogens detected in molecular testing is provided in Supplementary Appendix Figure 1. Cases with confirmed RSV positive with and without co-detection of non-RSV pathogens were included in the analysis as one group (RSV-positive group).

The hospitalization rate was higher among RSV-positive patients (87%) vs RSV-negative (77%). The median length of stay among all inpatients, regardless of RSV test status, was 3 days (interquartile range [IQR] 2, 4). About 60% of total participants (259) sought care prior to the index visit, including 55 of the 78 (73%) who tested positive for RSV and 126 of 202 (62%) who did not. The median monthly income among households seeking care prior to the index visit was less ($55.56) than the group that did not ($92.60). Participant characteristics are provided in Table 1.

**Table 1.** Participant Characteristics

	All infants (N = 426)		RSV positive (N = 78)		RSV negative (N = 202)	
	Inpatient	Outpatient	Inpatient	Outpatient	Inpatient	Outpatient
N (%)	320 (75%)	106 (25%)	69 (88%)	9 (12%)	156 (77%)	46 (23%)
Female	113 (35%)	46 (44%)	23 (33%)	2 (22%)	59 (38%)	20 (43%)
Age, months (mean, SD)	5.9 (3.34)	6.07 (3.1)	5.59 (3.46)	5.24 (3.23)	5.62 (3.46)	5.85 (3.28)
Median monthly household income, in 2018 USD	47	68	55	83	45	61
Median household size (IQR)	5 (4, 6)	4 (3, 6)	5 (3, 7)	4 (3, 4)	5 (4, 6)	4 (3, 5)
Mortality between hospital visit and follow-up, N (%)	12 (4%)	3 (3%)	1 (1%)	0 (0%)	6 (4%)	0 (0%)
Median length of stay, bed nights (IQR)	3 (2, 4)	0 (0, 0)	3 (2, 4)	0 (0, 0)	3 (2, 4)	0 (0, 0)
Received care prior coming to the hospital, N (%)	236 (74%)	23 (22%)	55 (80%)	2 (22%)	115 (74%)	11 (24%)

Abbreviations: RSV, respiratory syncytial virus; USD, US dollars; IQR, interquartile range; SD, standard deviation.

### Cost Per Episode of Illness

Total cost per episode of illness, by hospitalization status and payer, is reported in Table 2. The mean cost per RSV episode was $62.26 for inpatients and $12.51 for outpatients. The health system cost accounted for 73%–74% of this cost, while households accounted for 26%–27%. For RSV-negative episodes, the mean cost for inpatients and outpatients was $68.42 and $11.78, respectively; the health system accounted for 82%–83% of this cost and to households the remaining.

**Table 2.** Summary of Cost (in 2018 USD) Per Acute Respiratory Illness Episode, by Hospitalization Status and Payer

	All infants (N = 426)		RSV positive (N = 78)		RSV negative (N = 202)	
	Inpatient (N = 320)	Outpatient (N = 106)	Inpatient (N = 69)	Outpatient (N = 9)	Inpatient (N = 156)	Outpatient (N = 46)
Health system costs						
Mean	55.78	7.84	45.37	9.26	55.85	9.74
Median	41.50	6.52	41.47	9.78	43.12	9.69
95% CI^a^	49.35-62.21	6.68-9.01	38.74-52.00	7.34-11.18	47.14-64.57	7.33-12.14
Household costs						
Mean	14.15	2.33	16.89	3.25	12.57	2.04
Median	10.09	0.67	8.35	1.11	10.07	0.75
95% CI^a^	11.94-16.37	1.63-3.03	7.89-25.89	0.10-6.41	10.95-14.19	1.19-2.89
Total costs						
Mean	69.93	10.17	62.26	12.51	68.42	11.78
Median	54.81	9.16	50.89	10.90	55.84	10.11
95% CI^a^	63.06-76.81	8.78-11.57	50.87-73.66	8.24-16.79	59.59-77.25	9.22-14.33

Abbreviations: RSV, respiratory syncytial virus; USD, US dollars.

^a^95% confidence interval (CI) of mean values. For reference, the 2018 USD midyear official exchange rate for select region/countries (1 USD = 0.847 Euro, = 101.302 Kenyan Shilling, = 10.458 Zambian Kwacha, and  = 4.585 Ghanaian Cedis) [8].

Among all-cause ARI cases (including both RSV positives and negatives), the mean cost per episode was $69.93 for inpatients and $10.17 for outpatients. The health system accounted for about 77%–80% of these costs, while the households accounted for the remaining 20%–23%.

On average, across all ARI cases, the health system costs accounted for roughly 80% of the total cost for inpatients and about 77% for outpatients. Inpatients had relatively higher health systems and household costs than outpatients. There was no statistically significant difference in cost per episode between the RSV-positive and all-cause ARIs infants.

### Cost to the Health System

Mean direct medical costs incurred by the health system are given in Table 3.

**Table 3.** Direct Medical Costs (in 2018 USD) Incurred by the Health System

	All infants		RSV positive		RSV negative	
	Inpatient (N = 320)	Outpatient (N = 106)	Inpatient (N = 69)	Outpatient (N = 9)	Inpatient (N = 156)	Outpatient (N = 46)
Drug cost						
Mean	8.56	2.26	2.97	1.62	9.57	2.75
Median	3.74	1.26	2.86	1.10	3.70	1.26
95% CI^a^	4.46-12.65	1.53-3.00	2.33-3.61	0.09-3.15	3.10-16.04	1.13-4.36
Lab cost						
Mean	16.84	3.17	16.78	5.23	18.19	4.58
Median	20.49	0	20.49	6.28	20.49	6.28
95% CI^a^	15.66-18.01	2.34-4.00	14.41-19.15	3.53-6.94	16.49-19.76	2.98-6.18
Staff cost						
Mean	21.23	1.34	17.85	1.34	19.61	1.34
Median	14.66	1.34	14.66	1.34	14.66	1.34
95% CI^a^	18.18-24.29	1.34-1.34	13.49-22.21	1.34-1.34	15.78-23.44	1.34-1.34
Facilities cost						
Mean	9.25	1.07	7.78	1.07	8.54	1.07
Median	6.39	1.07	6.39	1.07	6.39	1.07
95% CI^a^	7.92-10.58	1.07-1.07	5.88-9.68	1.07-1.07	6.88-10.88	1.07-1.07
Total cost						
Mean	55.78	7.84	45.37	9.26	55.85	9.74
Median	41.50	6.52	41.47	9.78	43.12	9.69
95% CI^a^	49.35-62.21	6.68-9.01	35.74-52.00	7.34-11.18	47.14-64.57	7.33-12.14

Abbreviations: RSV, respiratory syncytial virus; USD, US dollars.

^a^95% confidence interval (CI) of mean values.

The mean direct medical cost to the health system was slightly higher among infants who tested negative for RSV (and the all-cause ARI infant group) as compared with the RSV-positive group ($55.85 vs $45.37 for inpatients and $9.74 vs $9.26 for outpatients). This difference was primarily driven by the differences in drug cost ($9.57 vs $2.97 for inpatients and $2.75 vs $1.62 for outpatients). Drug costs account for about 7% of the health system cost for RSV-positive inpatients and about 17% for RSV-positive outpatients. In general, staff and laboratory costs were the major drivers of inpatient cost, whereas drugs and laboratory costs were the drivers of outpatient cost.

### Cost to the Households

Roughly, 20% of the total cost per episode of illness was borne by households. For RSV-positive cases, mean households costs were $16.89 (median $8.35) for inpatients and $3.25 (median $1.11) for outpatients (see Table 4). We found no substantial difference in household cost between the RSV-positive, RSV-negative, or all-cause ARI groups.

**Table 4.** Household Costs (in 2018 USD) Associated With an Episode of Illness

	All infants		RSV positive		RSV negative	
	Inpatient (N = 320)	Outpatient (N = 106)	Inpatient (N = 69)	Outpatient (N = 9)	Inpatient (N = 156)	Outpatient (N = 46)
Direct medical cost prior to index visit						
Mean	0.81	0.19	1.10	0.12	0.66	0.16
Median	0	0	0	0	0	0
95% CI^a^	0.60 to 1.02	0.04 to 0.33	0.57 to 1.63	–0.16 to 0.41	0.40 to 0.92	–0.01 to 0.33
Direct nonmedical cost of index visit						
Mean	6.39	0.49	6.42	0.36	5.93	0.51
Median	4.02	0	3.43	0.28	3.89	0
95% CI^a^	5.63 to 7.16	0.21 to 0.78	4.19 to 8.65	0.16 to 0.56	4.95 to 6.92	0.07 to 0.96
Transportation						
Mean	5.39	1.03	5.32	0.41	4.83	0.86
Median	3.70	0.46	2.27	0.42	3.24	0.46
95% CI^a^	4.68 to 6.09	0.43 to 1.62	3.34 to 7.30	0.21 to 0.60	3.93 to 5.72	0.11 to 1.61
Subsistence and others						
Mean	1.21	0.07	1.27	—	1.29	0.05
Median	0.59	—	0.41	—	0.70	—
95% CI^a^	1.01 to 1.42	−0.01 to 0.14	0.74 to 1.80	—	0.99 to 1.59	−0.04 to 0.14
Indirect cost due to index visit						
Mean	6.83	1.40	9.28	2.25	5.94	1.14
Median	3.70	0.42	2.78	0.56	4.63	0.37
95% CI^a^	4.85 to 8.81	0.89 to 1.91	0.60 to 17.95	–0.97 to 5.48	5.07 to 6.80	0.57 to 1.71
Direct medical cost after index visit						
Mean	0.12	0.25	0.10	0.51	0.04	0.22
Median	0	0	0	0	0	0
95% CI^a^	0.03 to 0.21	0.04 to 0.46	–0.06 to 0.25	–0.67 to 1.70	–0.02 to 0.10	–0.12 to 0.57
Total household costs						
Mean	14.15	2.33	16.89	3.25	12.57	2.04
Median	10.09	0.67	8.35	1.11	10.07	0.75
95% CI (mean)	11.94 to 16.37	1.63 to 3.03	7.89 to 25.89	0.10 to 6.41	10.95 to 14.19	1.19 to 2.89

Direct medical costs include expenses on consultation, drugs, tests, or any other financial costs. Direct medical costs for an index visit were all covered by the health system and thus not included here. Indirect cost includes the lost household income due to taking care of sick children.

Abbreviations: RSV, respiratory syncytial virus; USD, US dollars.

^a^95% confidence interval (CI) of mean values.

Households incurred no direct medical costs from the health care visit, which was completely covered by the government. Pre- and/or post-index visit care accounts for roughly 7% of the total household cost. In general, households seeking care prior to the index visit incurred almost twice the household cost as those who did not.

**Figure 2.**
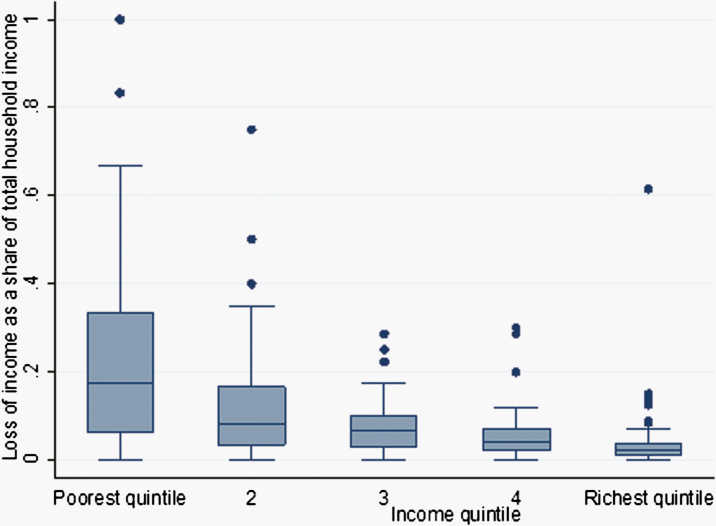
Loss of income as a proportion of total household income among families of inpatient cases. Monthly income quintile, poorest quintile (<$38); 2 ($38-$56); 3 ($57-$83); 4 ($90-$130); richest quintile (>$130). Numbers in the y-axis represents different income quintile. The horizontal bar within each box represents the median values within each sub group. The longer the box, the more dispersed is the data. In other words, the reported loss of income as a share of total household income among the poorest income group is much higher than that for the others.

Direct nonmedical costs from the index visit such as transportation and subsistence account for roughly 38% and 45% of the total household cost among RSV-positive and all-cause ARI inpatient episodes. Indirect costs due to lost income, as reported by parents, account for roughly half of the household costs across all groups (Table 4). A pairwise comparison of mean difference test shows the loss of income as a proportion of total monthly income was higher among the households in the poorest quintile. Figure 2 shows the proportion of income lost as a share of total household income by income quintile for families with inpatient children. The median loss of income as a share of household income is much higher among families in the lowest income quintile, and the proportion consistently declines as family income increases. Specifically, for inpatient families at the highest income level (>$95.5 per month), this loss represents about 4% of total household monthly income, but for inpatient families at the lowest income level (<$23.3 per month), it is about 32% of the total household income.

## Discussion

Cost data for infant ARIs are sparse in low-income countries and almost nonexistent for pathogens such as RSV, which disproportionately impacts young infants [10]. While the economic impact of RSV has been estimated [11], current evaluations have relied on costs of other pathogens or imputed RSV costs based on assumptions. This is one of the first studies to evaluate RSV costs specifically from a low-income country in Africa where we estimate the economic burden of RSV, as well as all-cause ARIs using a combination of patient/household survey data, patient chart review, and hospital administrative data. We tested a subset of patients with ARI to confirm RSV etiology and compare cost estimates among patients with and without RSV infection. More than 75% of the study subjects were hospitalized (inpatients) partly because the study was conducted in a referral hospital where severe cases are more likely to visit in general and more importantly, the study was nested within the SARI surveillance program.

The total cost per episode for RSV-associated ARI was 5 times higher for inpatients than for outpatients (Table 2). For all-cause ARI, which includes RSV-positive, RSV-negative, and non-tested cases, cost per episode was 7 times higher for inpatients than outpatients (Table 2). Although the absolute cost for an RSV-positive inpatient episode is less than that for an RSV-negative or all-cause ARI case, we found no substantial difference in total cost per episode between these groups. Across all illness groups, the median cost per episode estimates are less than the mean values, reflecting positively skewed cost data with few cases incurring very high costs.

Health system costs accounted for roughly 80% of the total cost per episode of illness. QECH paid all direct costs incurred during treatment (including cost of drugs dispensed). Staff and facilities followed by laboratory tests and procedures drove inpatient costs across all illness groups. The mean cost of drugs was roughly 3 times lower among the RSV-positive inpatients compared with RSV-negative or all-cause ARI inpatients (although the difference in median values was modest). However, our data suggest that RSV-positive patients were prescribed antibiotics less frequently than RSV-negative and other patients, possibly explaining the lower drug costs. Only 73% of RSV-positive patients were prescribed at least 1 antibiotic compared with 85% in the all infants group (see Supplementary Appendix Table 3).

Overall, household costs made up about 20% of the total cost per episode, almost half of which were due to reported lost income. Households in the poor income category lost up to one-third of their total monthly income to an RSV inpatient episode—a substantial share in a subsistence economy. Although households in Malawi were spared from shouldering a majority of direct medical costs associated with the index visit at the hospital, roughly 60% of cases sought prehospital care prior to the index visit (including traditional healers and self-medication), and this contributed to roughly 6% of all household costs. Prehospital care-seeking was even higher among the RSV-positive subgroup (73% on average). Households in the lowest income quintile tended to seek more prehospital care.

Although the number of cases positive for RSV only (with no codetection of other non-RSV pathogens) is small (N = 11), the difference in cost estimates observed among this subgroup compared with all RSV test-positive group is worth mentioning. The subgroup that was positive only for RSV virus (inpatients, N = 10) had about 65% higher costs per episode ($100.59) compared with the all RSV-positive subgroup ($62.26). The difference was more prominent for household costs (more than 130%) than for the health system costs (about 33%). The higher health system cost was driven by the staff and facility costs as the average length of stay among this subgroup was higher (6.2 days vs 3.65 days). The small number of RSV-positive subjects with no other non-RSV pathogens of significance detected is small and limits our ability to make inferences on costs of coinfection but makes an interesting subject for future research.

There are only a handful of studies from other low- and middle-income (LMIC) nations that present ARI costs, and even fewer that present costs associated with RSV in Africa, to which we can compare results. In Kenya, the mean cost of hospitalization for pneumonia is estimated at $177.14 (national hospital) and $62.08 (district hospital) [12]. In Zambia, the health system cost per outpatient and inpatient episode for children under the age of 5 with pneumonia was reported at $48 and $215, respectively [13]. In India, the total direct cost of an ARI episode for a hospitalized child ranged between $54 and $135 [14]. A more recent systematic review on the cost of managing severe pneumonia across LMICs estimated $51.70 for outpatients and $242.70 for inpatients [10].

The cost estimates generated in our study are much lower when compared with other countries in the region. This could be due to the general low-income country status of Malawi. The cost of each ARI hospitalization in Malawi is about 18% of its GDP per capita; for comparison, in Kenya, it ranges between 6% and 13%, depending on the type of hospital [12]. Further, in Kenya, 80%–83% of total costs are attributed to staff and facilities followed by investigations (11%-12%) and drugs (5%-9%). In Malawi, only 55% of the direct medical costs per ARI episode are attributed to staff and facilities. Lower staff salaries and facility costs may explain the relatively lower cost estimates in Malawi.

A previous study conducted in Malawi, which included data from the same study hospital as well as one additional health facility in a rural setting, estimated the cost of acute diarrhea in children and reported $47.21, $25.26, and $73.78 as the mean cost to the health system, household, and total cost per inpatient episode, respectively [15]. While the treatment and management of diarrheal and acute respiratory diseases may not be comparable, the cost estimates for RSV positives from our study are similar—$45.37, $16.89, and $62.26 for the health system, household, and total costs for inpatient episode, respectively.

An important finding from this study is that the costs between all-cause ARIs and RSV do not seem all that different, although more evidence is needed to definitively establish the differences.

This study has some limitations. Financial constraints limited our ability to conduct molecular testing on all study subjects. The sample size for RSV-positive cases approached the minimum requirement (78 compared with 80). Although samples were randomly drawn for molecular testing, testing the full set would have allowed for greater reliability and precision of results. Given that the study collected data from a tertiary-level hospital and was nested within a severe acute respiratory surveillance platform, a higher rate of severe cases of ARI were enrolled. Also, this study only includes costs for patients who sought treatment; however, there is a substantial community burden of ARI among children—especially RSV—who may not attend health facilities [16]. The estimates, therefore, may not fully account for the costs associated with unidentified and untreated community cases and the long-term consequences of these illnesses [17]. Prehospital and posthospital visit costs to the households were based on primary caregiver recall, and the estimates may be subject to potential recall bias. Lost income was only measured at the index visit, but we believe this encompasses the majority of income lost due to the proportionally lower costs of care to the household for prehospital and posthospital visits. Prehospital and posthospital visits are usually time caregivers spend at home with the patient with minimal interruptions in daily activities until when the illness is severe enough to warrant going to a health center. Most of the productivity losses are, therefore, around the time when they interface with health facilities. Further, household income level was based on reported monthly income and its measurement may include inherent reporting biases [19].

The study uses data from a single referral hospital in Malawi that provides free care to Blantyre’s urban and peri-urban populations, and findings may not be generalizable across differing healthcare settings. For example, the finding of modest absolute household costs may not apply in locations where households usually share the direct medical cost of hospital treatment. In many LMICs in Asia and Africa, basic health services are not free, and care represents a substantial financial burden for households. Malawi has made a concerted effort to make care free for children as a part of its essential healthcare package [18]. 

## Conclusions

This study demonstrates the substantial economic impact RSV and other respiratory illnesses can have on households and health systems in low-income settings. These costs are lower than reported in other studies that examined ARI costs. We believe this may be explained by the relative income status of Malawi and comparator countries; however, more information on RSV-associated costs in other settings is warranted. This study also suggests the costs associated with an episode of RSV are comparable to RSV-negative and other ARIs among infants of the same age. Future studies are needed to confirm whether these insights hold in other settings. Moreover, interventions for RSV and other ARIs, such as vaccines and monoclonal antibodies, are in development [20]; the estimates generated by this study will be important for evaluating the cost-effectiveness of such interventions and supporting governments and policymakers in identifying good value investments for improving health. Furthermore, these cost data are critical for calculating the full value of potentially avertable burden by vaccination. The cost estimates from this study should be considered by key stakeholders as part of an evidence package when RSV preventive measures are available.

## Supplementary Data

Supplementary materials are available at the *Journal of the Pediatric Infectious Diseases Society online* (http://jpids.oxfordjournals.org). 

piaa157_suppl_Supplementary-Figure-S1

piaa157_suppl_Supplementary-Materials

## Notes

***Acknowledgments***. The authors would like to acknowledge the support provided by the following individuals during the planning, data collection, and processing phases of this study: Themba Mhango and Laura Newberry from the Queen Elizabeth Central Hospital; Amelia C. Crampin and Hazzie Mvula from the Malawi Epidemiology Research and Intervention Unit, London School of Hygiene & Tropical Medicine; Charles Mwansambo from the Ministry of Health, Malawi; Nigel A Cunliffe from the Centre for Global Vaccine Research, Institute of Infection and Global Health, University of Liverpool; and Robert S Heyderman from the University College London. We would also like to thank Richard W. Lee for his assistance in processing the drug costs data and Katie Regan for reviewing this manuscript.

***Potential conflicts of interest***. All authors declare no conflict of interest to declare.

## References

[1] Troeger CE , Blacker BF, Khalil IA, et al.; GBD 2017 Influenza collaborators Mortality, morbidity, and hospitalisations due to influenza lower respiratory tract infections, 2017: an analysis for the Global Burden of Disease Study 2017. Lancet Respir Med 2019; 7(1):69–89. 30553848 10.1016/S2213-2600(18)30496-X PMC6302221

[2] O’Brien KL , Baggett HC, Brooks WA, et al; The Pneumonia Etiology Research for Child Health (PERCH) Study Group Causes of severe pneumonia requiring hospital admission in children without HIV infection from Africa and Asia: the PERCH multi-country case-control study. Lancet 2019; 394:757–79. 31257127 10.1016/S0140-6736(19)30721-4 PMC6727070

[3] Shi T , McAllister DA, O’Brien KL, et al. Global, regional, and national disease burden estimates of acute lower respiratory infections due to respiratory syncytial virus in young children in 2015: a systematic review and modelling study. Lancet 2017; 390:946–58. 28689664 10.1016/S0140-6736(17)30938-8 PMC5592248

[4] Troeger C , Forouzanfar M, Rao PC, et al.; GBD 2015 LRI collaborators Estimates of the global, regional, and national morbidity, mortality, and aetiologies of lower respiratory tract infections in 195 countries: a systematic analysis for the Global Burden of Disease Study 2015. Lancet Infect Dis 2017; 17:1133–61. 28843578 10.1016/S1473-3099(17)30396-1 PMC5666185

[5] Peterson I , Bar-Zeev N, Kennedy N, et al Respiratory virus-associated severe acute respiratory illness and viral clustering in Malawian children in a setting with a high prevalence of HIV infection, malaria, and malnutrition. J Infect Dis 2016; 214:1700–1711. 27630199 10.1093/infdis/jiw426 PMC5341080

[6] VacSurv The VacSurv Programme. Accessed May 19, 2020 https://www.mlw.mw/index.php/microbes-immunity-and-vaccines-theme-projects-profiles/323-vacsurv.html

[7] The World Bank Data. Inflation, consumer prices (annual %) Accessed May 19, 2020 https://data.worldbank.org/indicator/FP.CPI.TOTL.ZG

[8] The World Bank Data. Official exchange rate (LCU per US$, period average) Accessed May 19, 2020 https://data.worldbank.org/indicator/PA.NUS.FCRF

[9] World Health Organization (WHO) Department of Immunization, Vaccines and Biologicals. Guidelines for estimating the economic burden of diarrhoeal disease, with focus on assessing the costs of rotavirus diarrhoea. Geneva, Switzerland: WHO; 2005 Accessed July 7, 2020 https://apps.who.int/iris/bitstream/handle/10665/69137/WHO_IVB_05.10.pdf?sequence=1&isAllowed=y

[10] Zhang S , Akmar LZ, Bailey F, et al Cost of respiratory syncytial virus-associated acute lower respiratory infection management in young children at the regional and global level: a systematic review and meta-analysis. J Infect Dis 2020; 222: S680–7. 10.1093/infdis/jiz683 32227101

[11] Li X , Willem L, Antillon M, Bilcke J, Jit M, Beutels P Health and economic burden of respiratory syncytial virus (RSV) disease and the cost-effectiveness of potential interventions against RSV among children under 5 years in 72 Gavi-eligible countries. BMC Med 2020; 18:1–16. 32248817 10.1186/s12916-020-01537-6 PMC7132892

[12] Ayieko P , Akumu AO, Griffiths UK, English M The economic burden of inpatient paediatric care in Kenya: household and provider costs for treatment of pneumonia, malaria and meningitis. Cost Eff Resour Alloc 2009; 7:3. 19161598 10.1186/1478-7547-7-3 PMC2640355

[13] Chola L , Robberstad B Estimating average inpatient and outpatient costs and childhood pneumonia and diarrhoea treatment costs in an urban health centre in Zambia. Cost Eff Resour Alloc 2009; 7:16. 19845966 10.1186/1478-7547-7-16 PMC2770026

[14] Peasah SK , Purakayastha DR, Koul PA, et al The cost of acute respiratory infections in Northern India: a multi-site study. BMC Public Health 2015; 15:330. 25880910 10.1186/s12889-015-1685-6 PMC4392863

[15] Hendrix N , Bar-Zeev N, Atherly D, et al; VacSurv Consortium The economic impact of childhood acute gastroenteritis on Malawian families and the healthcare system. BMJ Open 2017; 7:e017347. 10.1136/bmjopen-2017-017347 PMC5589001 28871025

[16] Caballero MT, Bianchi AM, Nuno A, et al. Mortality associated with acute respiratory infections among children at home. J Infect Dis 2019; 219:358–64.

[17] McCollum ED , Nambiar B, Deula R, et al. Impact of the 13-valent pneumococcal conjugate vaccine on clinical and hypoxemic childhood pneumonia over three years in Central Malawi: an observational study. PLoS One 2017; 12:e0168209. 28052071 10.1371/journal.pone.0168209 PMC5215454

[18] Abiiro GA , Mbera GB, De Allegri M Gaps in universal health coverage in Malawi: a qualitative study in rural communities. BMC Health Serv Res 2014; 14:234. 24884788 10.1186/1472-6963-14-234 PMC4051374

[19] Clewwe P Measurement error bias in estimates of income and income growth among the poor: analytical results and a correction formula. Econ Dev Cult Change 2007; 56:163–189.

[20] PATH. RSVvaccine and mAb snapshot [Internet]. Seattle, WA: PATH; Published 2020 Accessed July 9, 2020 https://vaccineresources.org/files/RSV-snapshot-2020_03_26_HighResolution_PDF.pdf

