# Supplementary figures and images for: Estimating the Economic Impact of Respiratory Syncytial Virus and Other Acute Respiratory Infections Among Infants Receiving Care at a Referral Hospital in Malawi
Source: J Pediatric Infect Dis Soc. 2020 Dec 21;9(6):738–45. doi: 10.1093/jpids/piaa157 (PMC7864144; doi:10.1093/jpids/piaa157)

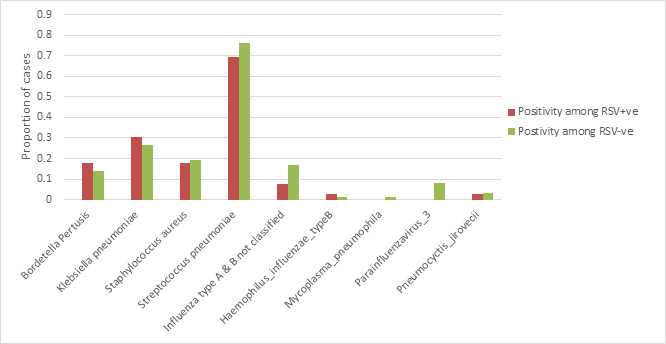

Supplement: piaa157_suppl_Supplementary-Figure-S1 [file piaa157_suppl_supplementary-figure-s1.png]
