# Supplementary material for: Estimating the Economic Impact of Respiratory Syncytial Virus and Other Acute Respiratory Infections Among Infants Receiving Care at a Referral Hospital in Malawi
Source: J Pediatric Infect Dis Soc. 2020 Dec 21;9(6):738–45. doi: 10.1093/jpids/piaa157 (PMC7864144; doi:10.1093/jpids/piaa157)
Supplement: piaa157_suppl_Supplementary-Materials [file piaa157_suppl_supplementary-materials.docx]

Supplementary materials

**Appendix Table 1: Unit cost of drugs**

| **Drug Name** | **Formulation** | **Route** | **Number of units per package** | **Units** | **Purchase price (MWK)** |
| --- | --- | --- | --- | --- | --- |
| Amoxicillin | Capsule | Oral | 1000 | capsules | 17648.97 |
| Amoxycillin | Suspension | Oral | 100 | ml | 450.00 |
| Amoxycillin | Suspension | Oral | 1 | vial | 407.04 |
| Benzathine Benzylpenicillin | IV | Injectable | 1 | vial | 247.19 |
| Benzylpenicillin | IV | Injectable | 1 | vial | 148.95 |
| Co-trimoxazole (Septrin) | Suspension | Oral | 1 | vial | 259.82 |
| Co-trimoxazole (Septrin) | Tablet | Oral | 1000 | tablet | 14425.57 |
| Gentamicin | Injectable | Injectable | 1 | vial | 43.07 |
| Ibuprofen | Tablet | Oral | 1000 | tablet | 7153.03 |
| Metronidazole | Injectable | Injectable | 1 | vial | 353.65 |
| Metronidazole | Suspension | Oral | 1 | vial | 369.54 |
| Metronidazole | Tablet | Oral | 1000 | tablet | 5432.33 |
| Nevirapine | Suspension | Oral | 1 | ml | 13.23 |
| ORS Sachet | Sachet | Oral | 1 | sachet | 162.89 |
| Paracetamol | PO | Oral | 1000 | mg | 395.00 |
| Paracetamol | PO | Oral | 100 | ml | 395.00 |
| Salbutamol Aerosol Inhalation | Inhaler | Inhalation | 1 | inhaler | 961.33 |
| Salbutamol Nebuliser Solution | Nebuliser | Inhalation | 30 | vial | 821.28 |
| Salbutamol | Tablet | Oral | 1000 | tablet | 2777.32 |
| Salbutamol Sulphate | Injectable | Injectable | 5 | vial | 35.60 |
| Zinc Sulphate | Tablet | Oral | 100 | tablet | 549.70 |

*Source: Central Medical Stores Trust, 2018*

**Appendix Table 2: Test utilization and unit costs of services**

| Test/investigations performed | % of patients receiving test | Unit price per test  (2018 MWK) |
| --- | --- | --- |
| Full blood count | 6% | 9,502 |
| Malaria parasite specimen (MPS) | 80% | 2,308 |
| Packed cell volume (PCV) | 76% | 2,288 |
| Electrolytes | 0% | 5,472 |
| Glucose | 5% | 5,472 |
| CSF | 3% | 20,139 |
| Blood culture | 46% | 10,408 |
| X-ray* | 69% | NA |
| NP swab* | 5% | NA |

**Note: We excluded the cost of laboratory tests and investigations performed as part of clinical research, as these are likely not reflective of costs incurred during standard clinical care.*

**Appendix Table 3: Proportion of patients prescribed with various drugs**

|  | At least 1 antibiotic prescribed | Amoxycillin | Benzylpenicillin | Cotrimoxazole | Gentamicin | Cloxacillin | Metronidazole | Salbutamol* | Zinc* | Ibuprofen* | Paracetamol* | ORS* | Nevirapine* |
| --- | --- | --- | --- | --- | --- | --- | --- | --- | --- | --- | --- | --- | --- |
| **All infants** | 85% | 58% | 54% | 9% | 47% | 2% | 1% | 18% | 4% | 1% | 87% | 2% | 0% |
| Inpatient | 84% | 53% | 71% | 9% | 61% | 3% | 1% | 24% | 4% | 1% | 85% | 2% | 1% |
| Outpatient | 87% | 76% | 4% | 10% | 4% | 0% | 2% | 1% | 2% | 1% | 93% | 2% | 0% |
| **RSV positive** | 73% | 46% | 56% | 4% | 35% | 3% | 1% | 15% | 3% | 3% | 85% | 1% | 1% |
| Inpatient | 74% | 43% | 64% | 3% | 41% | 3% | 1% | 17% | 1% | 3% | 84% | 0% | 1% |
| Outpatient | 70% | 60% | 0% | 10% | 0% | 0% | 0% | 0% | 10% | 0% | 90% | 10% | 0% |
| **RSV negative** | 83% | 58% | 54% | 10% | 50% | 2% | 0% | 22% | 4% | 0% | 83% | 3% | 0% |
| Inpatient | 82% | 52% | 68% | 10% | 64% | 3% | 0% | 27% | 4% | 1% | 81% | 3% | 1% |
| Outpatient | 87% | 78% | 4% | 9% | 4% | 0% | 0% | 2% | 4% | 0% | 91% | 4% | 0% |

*Note: * Not an antibiotic.*

**Appendix Figure 1: Proportion of non-RSV pathogens detected during molecular testing among confirmed RSV positive (N=78) and RSV negative (N=265)***

**Of the 280 samples who underwent molecular testing, 265 (95%) of the patients were tested for other pathogens. All those who did not undergo tests for other pathogens tested negative for RSV.*
